# Multifamily Therapy for Adolescents With School Refusal: Perspectives of the Adolescents and Their Parents

**DOI:** 10.3389/fpsyt.2021.624841

**Published:** 2021-06-10

**Authors:** Aurélie Roué, Aurélie Harf, Laelia Benoit, Jordan Sibeoni, Marie Rose Moro

**Affiliations:** ^1^Maison des Adolescents—Maison de Solenn, Hôpital Cochin, APHP, Paris, France; ^2^Faculty of Psychology, Medical School, PCPP, University of Paris, Boulogne Billancourt, France; ^3^Center for Research in Epidemiology and Population Health, Paris-Sud and UVSQ Medical Schools, French National Institute of Health and Medical Research (Inserm), Team DevPsy, Villejuif, France; ^4^Service Universitaire de Psychiatrie de l'Adolescent, Argenteuil Hospital Centre, Argenteuil, France; ^5^ECSTRA Team, UMR-1153, Inserm, Paris University, Paris, France

**Keywords:** school refusal, multifamily therapy, qualitative research, adolescents, family therapy, school phobia, group therapy

## Abstract

**Introduction:** School refusal is an important public health concern in adolescent psychiatry increasing over the past several years (5% of child and adolescent psychiatry consultations in France). Multifamily therapy has developed over 30 years. Its efficacy is validated in adult, child and adolescent psychiatry, including for children at risk of school exclusion. In this study, we aimed to explore the adolescents and their parent's experience of a multifamily therapy treatment of school refusal with a qualitative method.

**Materials and Methods:** This qualitative study is based on an Interpretative Phenomenological Analysis approach. We conducted 15 semi-structured interviews, participants were adolescents (*n* = 6) and their parents (*n* = 9) who experienced multifamily therapy in an adolescent department in Paris. Data analysis was performed independently by two researchers.

**Results:** For the six families, school was a source of suffering, system paralysis and social exclusion. Families reported painful emotions and separation anxiety. For teenagers, multifamily therapy increased self-confidence and allowed group experience. For parents, it gave support and relieved from feelings of stigmatization and guilt. Parents became more aware of their adolescent's suffering and their insight. They all considered that multifamily therapy improved intra-family communication and expression of emotion. Participants highlighted the benefits of intergenerational interactions, activities, group and guidance from therapists.

**Discussion:** Multifamily therapy uses therapeutic tools from both family therapy (joining, resonance, family competence, and metacommunication) and group therapy (use of media, identity device, and mirror reactions). Parents expect school solutions from multifamily therapy and question how psychiatric treatment can deal with school, school refusal being therefore understood as a social functioning disorder.

## Introduction

### School Refusal, a Major Public Health Issue

School refusal is a worldwide current public health issue (1, 2). It increased over the past years and concerns 5% of consultations in preadolescence and adolescence psychiatry in France (3–5). First named “school phobia” by (6), school refusal is distinct from others forms of school attendance problems (SAP), such as truancy, school withdrawal, and school exclusion. In truancy, reasons and motivations of school missing are different (positives rewards). Anxiety is not present, and absenteeism is frequently hidden from parents. Young people are not willing to go to school. Finally, truancy, as school exclusion, are more often associated with behavioral disorder and oppositional defiance disorder (2). Berg's consensual international definition of school refusal relies on four criteria which are the following ones (7). The young person is reluctant or refuses to attend school, in conjunction with emotional distress that is temporal and indicative of aversion to attendance (e.g., excessive fearfulness, temper tantrums, unhappiness, unexplained physical symptoms) or emotional distress that is chronic and hindering attendance (e.g., depressive affect; sleep problems), usually but not necessarily manifested by absence (e.g., late arrivals; missing whole school days; missing consecutive weeks, months, or years). The young person does not try to hide associated absence from their parents (e.g., they are at home and the parents are aware of this), and if they previously hid absence then they stopped doing so once the absence was discovered. The young person does not display severe antisocial behavior, beyond resistance to parental attempts to get them to school. Finally, the parents have made reasonable efforts, currently or at an earlier stage in the history of the problem, to secure attendance at school, and/or the parents express their intention for their child to attend school full-time. The existence of reasonable parental efforts is important to assess school refusal. Neither DSM 5 (Diagnostic and Statistical Manual of Mental Disorders) nor ICD-10 (International Classification of Diseases) recognize school refusal as a diagnosis, underlying the social and educational issues at stake.

The diagnosis is done on clinical criteria and can be assessed by the School Refusal Assessment Scale (SRAS) developed by (8). School refusal can be divided into different subcategories: school refusal associated with anxiety separation, school refusal associated with anxiety (including social anxiety), school refusal associated with specific phobia related to school (tests, teachers), school refusal related to depression (1). School refusal concerned two main ages: childhood (first primary classes) and adolescence. Prevalence in adolescents appear to be higher than in childhood (1). Prevalence does not vary according to sex, socioeconomic background or intellectual level. Half of school refusal appear in the form of somatic complains (headaches, abdominal pains, nausea, sleeping disorders) (1, 9).

School refusal is associated with several comorbidities. Half of patients suffer from anxiety and depressive disorders (10). Short term consequences are weak academic performances (31%), impacts on peer relations (34%), family conflicts (43%), school leaving (25%) exclusions from peers, risky behaviors (addiction), and suicide attempts (11–14). Thirty to 50% of these adolescents still have psychiatric disorders when they are adult (anxiety, depression, personality disorders) (6, 15). They stay longer at their parents', have less children, consume more psychiatric care and suffer from more professional failure (16). As a result, some authors state that back to school is only one prognostic element among others according to the ability of general adaptation (17).

Thus, school refusal is a major issue and its treatment is a priority. Early intervention is required, and prognosis depends on how much school the child misses (1). The first objective is the back to school. According to the school refusal severity, adolescents can benefit from outpatient cares, a day hospital or a complete hospitalization. Cares are multidisciplinary. An individual psychotherapy is always proposed- cognitive-behavioral therapy (CBT) has shown positive outcomes (18). Medication is proposed when required (anxiolytic or antidepressant) (6). Working with families and school is also essential.

### Family Involvement in School Refusal Treatment

Working with families appears to be a crucial element in child and adolescent psychiatry (19). School refusal has a major impact on families, and may stretch the parents' vulnerability (20, 21). Moreover, the degree of commitment of school personnel toward children with school refusal largely depends on their parents' attitudes (22). The first family therapy for school refusal is described by (23, 24). Several studies have since evaluated and confirmed the efficacy of family adjunction in therapies (12). In their qualitative study about the Experience of Psychiatric Care of Adolescents with School Refusal (25), Sibeoni et al. showed that expectations were different between adolescents and parents. Adolescents considered their suffering as the principal difficulty—suffering which parents, relatives and even health professionals could underestimate. School was not seen as the source of their discomfort—rather the place where that discomfort could be expressed. On the contrary, parents focused on their children being back to school which remained the central issue- psychiatric care had to resolve this difficulty. They worried about their child's future in school, repetition and school system rigidity. They were also worried about their child's social isolation, seeing the hospital as a mean of sociability. Parents perceived their child's internal discomfort at a later stage and sometimes after professional explanations. In addition, they attributed their child's progress during treatment (improvement in psychiatric disorders, better self-confidence, and maturity) to the development of adolescence more than to psychiatric care. Intra-family and peer relationships was also emphasized, as adolescents may say that changes in family relationships were beneficial. Thus, expectations of parents and adolescents differed. On one hand, adolescents focused on taking charge of their internal discomfort and the importance given to time, while parents were focused on the return to school- being synonymous with recovery-, and the need to find quick solutions. As a result, involving families in school refusal treatment appears to be quite challenging. Family adjunction improve therapeutic outcomes including long term outcomes (especially for behavior and cognitive therapy) (12, 26–29). Carr showed that family therapies improved symptoms (anxiety and school leaving) in more than 2/3 of school refusal patients, thus being more efficient than individual therapy (30, 31).

### Multifamily Therapy

Multifamily therapy (MFT) first appeared in United States in the 1960's (32). Based on family and group approaches, MFT proposes to several families, who have a member affected by the same disease—originally schizophrenia—to help each other in a “caring community (33, 34).” Through the exchange of ideas and experiences with other relatives and members of other families, participants can compare notes and learn from one another (34). Intra- and inter-family interactions are “intensified” (35) in a group setting where parents and children are participating in different exercises. Participants not only examine their own interactions but also those of others families and their individual members. The creation of multiple perspectives, which is much harder to replicate in individual sessions, is associated with change (36). MFT has largely spread these last 30 years and has gained empirical supports with no clear contraindications (37). It is currently used in adult psychiatry and in child and adolescent psychiatry (e.g., for ADHD, learning disorders and others) (37–41). It has been well-described that MFT is as efficient as and less costly than family therapy for anorexia nervosa. It has also been used for social issues, such as for families with abused children. In 1977, Dawson and McHugh developed the “Family School” in the Malborough Hospital department. The “Family School” gathers several children at risk of exclusion from school and their families and teachers, using MFT (42, 43). Its efficacy was validated by Morris et al. (36). They found positive effects on child and family social, emotional and behavioral functioning. These effects lasted up to 12 months after the end of the therapy. Thus, MFT seems to be an interesting therapy for school refusal (36).

### The Present Study

To our knowledge, no international nor national studies have evaluated multifamily therapy in school refusal. Moreover, few data are available on the perspectives of adolescence and their parents on their experiences of school refusal and family therapy. The objective of the study was to explore experiences of a MFT treatment of school refusal among adolescents and their parents. We aimed to study the expectations, the lived experiences, the improvement and the critics and unfulfilled expectations. We wondered if MFT had an impact on school refusal and more broadly on individuals and on intra family interactions. To this aim, we chose a qualitative study design based on interpretative phenomenological analysis. Indeed, qualitative methods seek to describe and understand in depth a complex phenomenon. They were a tool of choice for focusing on the views of patients, including adolescents and families (25, 44).

## Participants and Methods

### Procedure

Since 2019, adolescent department of Cochin hospital has been providing a MFT program to adolescents attending the department for school refusal and their families.

This MFT program was based on five sessions, each one lasted 3 h, took place once a month and gathered 5–7 families. There were 4 therapists, trained in systemic family therapy—APRTF (Paris Association for Research and Work on Families), and in MFT by Asen (33).

The program content was developed by the Multifamily Therapy Team, supervised by Cook-Darzens (45) and Asen, based on the specific needs of families of adolescents with school refusal.

This MFT program had 6 main goals:

- improving anxiety manifestations to help adolescents going back to school,
- avoiding chronic school dropout,
- improving communication between adolescents and their families,
- developing the skills and resources of families,
- fostering a place for exchange for families facing the same problem,
- and breaking the feeling of isolation of families.

The content of the five sessions was manualized. For each session, the objective of the session and several alternative exercises were specified.

#### Sessions Description

Each session began with a sharing of the participants' mood of the day, then began the first part of the session. Activities could take place in the whole group or in parents, mother, father or adolescent subgroups. They could use different media, such as photos, drawings, role play, sculptures or speech. Feedback took place at the end of the activity, in whole group or in sub-groups. A break marked the end of the first part. The second part proposed another activity and ended with a feedback of the whole group session.

Each session worked on a theme:

- 1st session: meeting and creating the group alliance (photolanguage, cross presentation)
- 2nd session: outsourcing and providing information on the school refusal (problem drawing, expert intervention)
- 3rd session: change motivations (sub-groups, social network map)
- 4th session: family resources (cross presentation, role plays)
- 5th session: working on change and focusing on the future and the family's resources (role-playing, sculpture).

We conducted an Interpretative Phenomenological Analysis (IPA) study among adolescents and their families of the first MFT group which occurred from May to August 2019.

### Ethical Standards

This study was carried out in accordance with the recommendations of an appropriate ethics review board (Inserm ethics review board, IRB 20151300001072). All patients and their parents provided written consent before inclusion in the study. The semi-structured interviews were all audio-digitally recorded and anonymized.

### Sampling and Participants

Sampling was exhaustive since all the participants of the first session agreed to be recruited. The inclusion criteria were families with an adolescent aged from 12 to 18 years, with school refusal resulting in complete school disconnection for more than 2 weeks and <18 months, attending the adolescent department for school refusal and living in Ile de France. Diagnostic was assessed in team according to the Berg criteria. Adolescents had all a DSM 5 diagnostic. They had no mental deficiency and no neurodevelopmental disorders (autistic disorders, learning disorders, behaviors disorders). They used to be in regular school with an average academic level.

### Data Collection

Data came from semi-structured interviews we performed at the Cochin adolescent department. Participants were contacted by email or by phone. Each family was interviewed and parents and adolescents were interviewed together. Each interview lasted from 60 to 90 min. They were conducted by two researchers (AR, an adolescent psychiatrist and CG, an adolescent psychologist) from September to November 2019. The interviewers used an interactive conversational style and sought to explore experience of the participants, their feedback on the content of the sessions and the proposed activities, as well as the perceived changes in the family system and for school refusal (Table 1). The interviews, which have been anonymized, were recorded and transcribed word-for-word, including the participants' expressive nuances. The transcript thus obtained was then analyzed.

**Table 1:** Interview guide.

1	Taking news on the current situation.
2	Can you describe your experience in the multi-family group?
3	What were the most common emotions and feelings (anger, sadness, anxiety, relief, joy, fed up, etc.)?
4	What moment(s) during the therapy was (were) the most memorable? Describe this or these moment(s).
5	What changes has multifamily therapy made possible?
6	What hasn‘t changed with multi-family therapy?
7	In the proposed activities, did any of them seem relevant (interesting)?
8	In the proposed activities, did any of them seem uninteresting or unsuitable?
9	In multi-family therapy, what moves the problem forward? (What are the elements that you find the most useful in moving forward with the problem?)
10	What were your expectations from multifamily therapy before starting the group?
11	Did multifamily therapy fulfill your expectations?
12	What changes would you suggest for the next groups?
13	How would you explain multi-family therapy to someone who hasn‘t participated in it?
14	Visual analog scale from 0 to 10 to describe whether they are happy (10) or not (0) with their experience.

### Analysis

We performed a thematic content analysis according to the principles of IPA (46). The IPA allows for an in-depth analysis of the subject's subjective perception and the meaning given to lived experiences. IPA has three principal epistemological underpinnings: phenomenological- to understand how a phenomenon appears in the individual's conscious experience—, hermeneutic- dual process in which the “researcher is trying to make sense of the participants trying to make sense of what is happening to them”—and idiographic—a deep understanding of each case from the perspective and within the context of the individual (46). In practice, five subsequent steps have been followed (Table 2) (47). Each interview was analyzed in detail, then a transversal analysis was carried out in order to develop the final themes and sub-themes and organize them. Data analysis was performed independently by two people (AR and AH, two adolescent psychiatrists) so that the themes identified did not reflect the unique vision of a single researcher. Triangulation of the analysis, which guarantees the quality of individual coding, took place during monthly meetings of our research group (AR, AH, LB, JS, and MM, all adolescent psychiatrist).

**Table 2:** The five steps of analysis data.

	**Activities**	**Rationale**
Stage 1	Repeatedly read each transcript, as a whole	Obtain a global picture of the interview and become familiar with the interviewee‘s verbal style and vocabulary.Each new reading of the transcript might also provide new perspectives.
Stage 2	Code the transcript by making notes corresponding to the fundamental units of meanings.	Make descriptive notes using the participant's own words.
Stage 3	Make conceptual notes through processes of condensation, abstraction, and comparison of the initial notes.	Categorize initial notes and reach a higher level of abstraction.
Stage 4	Identify initial themes. Provide text quotes that illustrate the main ideas of each theme.	Themes are labels that summarize the essence of a number of related conceptual notes. They are used to capture the experience of the phenomenon under study.
Stage 5	Identify recurrent themes across transcripts and produce a coherent ordered table of the themes and sub-themes.	Move from the particular to the shared across multiple experiences. Recurrent themes reflect a shared understanding of the phenomena among all participants.During this more analytic stage, researchers try to make sense of the associations between the themes found.

## Results

### Sample

This study included 15 participants who attended the first session: 6 adolescents, 6 mothers and 3 fathers. Five of the 6 adolescents were girls. Half of the parents were together. All the adolescents had fully left school at least since 5 months. They all had psychiatric co-morbidities (anxious and depression disorders) and they all had psychiatric care. Table 3 summarizes their characteristics.

**Table 3:** Participants' characteristics.

	**Family 1**	**Family 2**	**Family 3**	**Family 4**	**Family 5**	**Family 6**
Age	15	16	16	15	17	14
Sex	F	F	F	F	F	M
Academic level Repetition/passing a class)	2^nde^ general	1^re^ STMG	1^re^ general	3^e^ general	1^re^ general arrangement with school then CNED	3^e^ general
Class dropout	4^e^ then in 2^nde^	2^nde^	3^e^	4^e^	2^nde^	3^e^
Class dropout duration	Some months in 4^e^ then arrangement with school ; full since 9 months	18 months	Part-time since 3 years; full since 6 months, with CNED	Part-time since 1 year and 5 months; full since 5 months	Since 1 year and a half; full since 5 months	Since 6 months
Harassment	Yes, in 4^e^		Yes, during school	Yes, during school		
School change	Current demand			Change between 4^e^ and 3^e^	In 2^nde^	
Next year plan	CNED	Bachelor's degree in 2 years	CNED	Repetition and CNED	Care and study hospital	
Social life	Preserved		Preserved (a boyfriend)	Social networks (a boyfriend)	Preserved	Any
Comorbidities	Anxiety Panic attack Major depression	Depression	THC addiction Scars Depression Anorexia nervosa	Scars Anorexia nervosa	Scars TAG Panic attack Depressive disorders Mix anorexia nervosa ADHD	Suicide ideas
Psychiatric history	CMP care in 4^e^	Psychological care at CMP during 7 months in 2018	Psychological care since 2017 then followed at MDA Hospitalization at MDA from May to June 2019	Psychopraticien in 2017	Hospitalization Day hospital from January to June 2018	
Family history	Brother: school anxiety Mother: depression		Father: anxious disorder		Mother: depression	Father: school leaving in 2^nde^ Depression
Current psychiatric history	Psychological care since September 2018 Adolescent psychiatric care	Psychotherapy CBT since April 2019 Adolescent psychiatric care	Adolescent psychiatric care	Day hospital since September 2018 Adolescent psychiatric care	Psychological care Adolescent psychiatric care Family therapy since April 2019	Psychological care
Treatment	Sertraline prescribed not taken	Deroxat 20 mg	Sertraline 100 mg	Any	Sertraline 150 mg	Any
Brotherhood	1 brother (18 years old) Brothers and sisters in law	2 brothers in law	1 brother (19 years old)	2 brothers in law	Single child	1 brother of 20 years
Parental situation	Parents together	Parents divorced	Parents together	Parents separated	Single	Parents together
Living place	At her parents' with her brother	At her mother's	At her parents' (brother doesn't live anymore)	At her parents'	At her mother's	At his parents' (brother doesn't live anymore at home)
MFT Participation	Parents and teenager	Mother and teenager	Parents and teenager	Mother and teenager	Mother and teenager	Parents, teenager, brother

*STMG, management science and technologies; CNED, national center of distance learning; CMP, medical and psychological center; THC, tetrahydrocannabinol; GAD, generalized anxiety disorder; MDA, house of adolescent; CBT, cognitive behavioral therapy*.

The results captures three superordinate themes:

1. Before: From School Refusal to MFT
2. The Living Experience of MFT
3. After MFT: Outcomes and Expectations

Table 4 summaries the thems and sub-themes.

**Table 4:** Summary of themes and categories.

**Themes**	**Categories**
Before: from School Refusal to MFT	Academic issues are major Families experience deep suffering and paralysis
	Stigmatization and search for identity
	Anxiety from school remains overwhelming
	These families experience great separation anxiety
The living experience of MFT	Description of the MFT device by the participants
	*Experiencing generational diversity safely*
	*Getting involved in activities*
	*Belonging to a group*
	*Therapist as a security figure*
	A Deeply Emotional Trip
	*Emotional catharsis*
	*Group connection*
	*Awareness*
After MFT: outcomes and unmet expectations	Empowered Families More liberated adolescents
	Breaking the Taboo of School Refusal
	Relationships with others
	Unmet expectations

Relevant quotations from the tran-scripts are presented within the results, they have been translated into English for the sole purpose of this article.

### Before: From School Refusal to MFT

#### Academic Issues Are Major

In these families, academic and social success issues seemed to be major. Grades were a central element with strong pressure, pressure from adolescents who inferred their intelligence to their academic level and pressure from parents (Q1). Success must be academic and academic failure was a loss for both parents and adolescents (Q2). Performance anxiety was strong (Q3). Teenagers described their parent's expectations as a heavy weigh to bear (Q4). School was perceived in very negative terms: a cemetery (Q5), a brake and a hindrance. Adolescents were frightened and experienced a deep unease at school (Q6). Schooling affected the family atmosphere, provoked misunderstandings (Q7) and family conflicts (Q8).

*Q1 Mother 5: “She came very happy with a math homework graded C. I looked at the column next to it, the class average was B and I didn't value that.”*

*Q2 Father 1: “She wants to succeed, and she has largely the capacity for. It would be a shame if she stayed by the side of the road”*

*Q3 Mother 1: “For her, repeating a year, that was a shame, it means she was stupid”*

*Q4 Teenager 5: “Because, suddenly, children would have in their heads the expectations that parents have of their children and that can put a lot of pressure.”*

*Q5 Father 6: “On the way to school, there was the cemetery [.]. Yes, in the drawing, they put hell in it.”*

*Q6 Teenager 3: “I was scared of high school and all that.”*

*Q7 Mother 5: “Teenager 5 thinks that, for a long time, I put pressure on her, real or unconscious, or that I have wanted to compare her to others.”*

*Q8 Teenager 3: “Let's say it was usually my daddy who came in with his big hooves, who asked me the question he shouldn't ask and suddenly I would go straight and it ended badly.”*

#### Families Experience Deep Suffering and Paralysis

School refusal generated violence, suffering, fear and hopelessness (Q9). It produced a deep feeling of helplessness and loneliness without support and without a concrete solution. Families described themselves as wandering and lost (Q10). It was a daily struggle (Q11). These families also experienced great guilt, searching what role they had in the school refusal development and what they had missed (Q12). Mothers described themselves as not enough good mother. Some parents had even not told their relatives about it (Q13).

*Q9 Teenager 5: “Yes, violence and even hatred that we can have inside us, frustration, something that is really present when we can't go to school, when we are not well [.] even parents.”*

*Q10 Mother 1: “As parents, we are lost. I mean lost, we have to research, we have to find […] we, parents, have to do everything.”*

*Q11 “Father 6: Here we faced a system and we defend ourselves.”*

*Q12 Mother 5: “I wonder, I search, how this situation happened, what are its causes, what part, what role I might have played”*

*Q13 Mother 3: “I didn't tell my parents anything. So it had been 2 or 3 years or so, I didn't say anything to my parents.”*

School refusal paralyzed daily life with no temporal landmarks anymore for teenagers (Q14). Families were stunned by fear and shock. The disease became all-powerful and tyrannical, locking and imprisoning the adolescent and his family (Q15). Paralysis hardened, no more moves were possible. Acting or speaking could worsen things.

The family system was paralyzed with no emotion shared (Q16). The family was divided by major differences between parents and adolescents experience, especially between parents stunned by anxiety and adolescents who experienced daily school refusal (Q17). Parents didn't understand their children behavior anymore, nearly with a feeling of lost.

Finally nothing happened no more, everything was frozen. Imagining a future was impossible (Q18). More than future, school refusal paralyzed the development and arose death anguish. Thus, death theme appeared throughout the interviews in a use of a vocabulary close to the act of execution or through metaphors: tunnel, drowning (Q19).

*Q14 Teenager 1:* “*I hadn't any reasons to wake up, I have nothing to do”*

*Q15 Mother 1: “And then I couldn't go because, as soon as I went outside for 5 min, she beeped me ‘Mummy, mummy, come back, come back, I have dark thoughts.' So, we left everything. Now it's okay I can go out.”*

*Q16 Mother 3: “And then we have to keep quiet. When we are in family meetings, we can't express ourselves, tell what we feel, because we have to resist for others. But that's what is difficult, because you can't show anything […]. And you build a wall.”*

*Q17 Teenager 3: “Parents arrive with their big hooves to tell us that and put it all in our heads, that totally ends the conversation […] it was not that we didn't want [.] it was really something we couldn't do.”*

*Mother 2: “I understand now how my daughter works and why we are here.”*

*Teenager 3: “I had the feeling that MFT was much more demanding for you, parents, than for us. We, about school refusal, we are already inside”*

*Q18 Teenager 5: “Imagining myself years later is something I can't think of.”*

Q19 Father 3: “It's true it was gut-wrenching for me […] skinned themselves.”

Mother 3: “We were at the bottom of the tunnel about everything, in a general sense.”

*Mother 1: “Finally, we take our heads out of the water […] there is no life belt.”*

#### Stigmatization and Search for Identity

Families experienced a strong societal and school stigmatization. School refusal was unknown and not recognized, and thus generated a triple exclusion. Exclusion from peers, pupils, parents, families (Q20). Exclusion and abandonment by the academic and teaching system (Q21). Exclusion by society and the state (Q22).

*Q20 Teenager 1: “With others pupils, we are often afraid of being judged because we know it's hard for them to understand the difference.”*

*Mother 3: “Because when we say ‘Don't go to school,' some people, who don't experience what we do, say “she is lax, they accept everything from their daughter!.””*

*Teenager 3: “There was mother 2, her family, they didn't get anything, they weren't understanding about what was happening and she was getting a lot head over heels about her daughter. Mother 1 too.”*

*Q21 Father 3: “That means that high school dropped us. Them, it's a situation they refuse, something they ignore.”*

*Q22 Mother 1: “Children like ours are misunderstood by the system and the society. These children are children who get into troubles.”*

Living this exclusion, families seemed to search for an identity that school denied them. They appeared to be ambivalent toward school refusal, hardly naming school refusal and using terms as “problem,” “problems,” “problematic,” “situation,” “particularity” (Q23). Likewise, depression and care were rarely named and difficulties were minimized (Q24). This ambivalence appeared in the participants expectations toward MFT: for adolescents, to be with others peers or to make their parents becoming aware; for parents, to find solutions for their children (Q25). Care did not appear in their expectations.

School refusal was a special way of being, which must be understood and supported. An enigma they were looking for (Q26). Their adolescent behavior even became a higher non-standard one. Thus, school refusal proved this identity. It wasn't their children who had a disorder, it was the academic system which didn't recognize and fit to their particular and gifted teenagers (Q27). School refusal offered them an identity to belong (Q28).

However, return to school still remained an ideal to achieve, identifying intelligence and adolescent normality, fitting thus a social norm as exemplified by the road metaphor throughout the research (Q29).

*Q23 “When you say ‘the problem', is it?*

*Mother 4: The anxious refusal of Teenager 4.”*

*Q24 Mother 2: “It's a disease in quotes”*

*Q25 Father 6: “The first thing, we did it for Teenager 6 […] to get better, finally a tool, to be better equipped, both to understand the origin of the problems and to correct problems”*

*Teenager 6: “Meet other people”*

*Q26 Mother 4: “It's a better understanding of this functioning […] Teenager 4 is overwhelmed by it.”*

*Q27 Mother 5 “[…] set up with a sophrologist, specialized in orientation for children with particularity, dys, ADHD, etc. […] we look for other possibilities since we have to step outside the box.”*

*Q28 Teenager 1: “School phobia is very specific [.] there are many others who are different like us.”*

*Q29 Teenager 1: “Well, I know where I'm going. I have a specific goal now.”*

#### Anxiety From School Remains Overwhelming

Schooling was overwhelming. Nothing existed apart school, families only spoke about school and couldn't speak of others topics. Even when they were asked for personal news, they answered with schooling (Q30).

*Q30 “And how are you at home?”*

*Mother 4: “Uh, it's okay. Well, it was a bit complicated with the CNED (national center of distance learning), it got me a little bit over* … *I think it still stress us out, bored and stressed us too [.] here we are, things are moving forward.”*

Nothing else could be thought. Painful emotions were avoided and couldn't be shared or showed in family (Q31). These families got difficulties to identify their emotion and to name them (Q32). Introspection was hard and some families preferred hiding behind science and theory more than experiencing emotions (Q33). They were affected by the emotion expressed by other participants but not by theirs (Q34). Parents described themselves as inhibited, avoiding oral situations where they could be in danger. For these aspects, MFT was frightening at the beginning (Q35).

*Q31 Mother 6: “I'm not saying it's good, but that's how it is. I consider that I don't have to let it go [my emotions].”*

*Q32 Father 3: “I had a lot of emotions, which I was trying not to show […]. It's the emotion of someone watching other scenes, I was embarrassed.”*

*Q33 Father 6: “So [.] to scientificize a problem which, at the beginning, is very emotional.”*

*Q34 Mother 3: “I find it hard emotionally because there were parents…. Above all about parents and even children.”*

*Q35 Mother 1: “Initially, I didn't really want to go, I didn't want to show my life and I don't like to talk about it”*

Adolescents appeared to be in great difficulties when they were asked to think, as if they preferred taking no risk to think or act by fear of being wrong. They were marked by a first astonishment of thought as soon as they were asked a question (Q36). Language production was difficult, even absent. They answered mostly with sentences as “I don't know.” Emotions were also difficult, whether their access, their identification or their naming (Q37). They were also adolescents marked by an excessive fear of other judgments and they were paralyzed by social anxiety. They didn't have confidence in themselves and were afraid of doing wrong. The unknown, especially the changes, scared them (Q38). They therefore needed security and landmarks.

*Q35 “Did you keep relationships after the group?*

*Teenager 6: I don't understand the question.”*

*Q36 Teenager 3: “When we say: ‘Things are not going on ‘or' I'm tired,' we have to develop it, to say why. Me, it makes me stuck”*

“*What were you feeling?”*

*Teenager 3: “I don't have any idea […]. It was pretty neutral.”*

*Q37 “What were you afraid of, do you know?”*

*Teenager 4: “Just the fact that it was new and that I didn't know.”*

#### These Families Experience Great Separation Anxiety

We noted on several occasions an absence of mother/daughter differentiation. This lack of differentiation even crept into the speech, where mothers intervened, added, supplemented, and thought for their adolescent (Q39). Mothers also seemed to be ambivalent toward adolescent's autonomy, both wanting them to be empowered and finding hard at the same time to let them empowered (Q40). Adolescents delegated their thoughts and words to their mother (Q41). This fusional mother-daughter relationship appeared ideal and idealized (Q42). There seemed to be no apparent problem in mother-daughter communication and the disorder brought them together and strengthened family ties (Q43).

Conflict was difficult, if not impossible. Thinking differently could mean rejection and exclusion (Q44). Identity exists only through fusion- fusion with others, peers, parents, adult referent (Q45). We could see it in the use of the pronominal “we” instead of “I.”

Separation with the therapists was difficult at the end of the session and parents expressed the feeling to be “drop out” (Q46). Nevertheless, relations between the participants didn't last after the end of MFT as if this idealized band couldn't exist outside of the therapy space (Q47).

*Q39 Mother 1: “She's always glued to mom, too.”*

*Q40 Mother 1: “There, it's still the little girl, my little baby, but no […]. By saying to myself: well no, they don't think like us.”*

Q41 Teenager 2: “It's not me you should ask, I wouldn't know at all how to explain, that's why when my mother says things I say it's all the same because that's exactly what I'm thinking so here's to you.”

*Q42 Teenager 5: “Yes! Just, I noticed once again during these sessions that we were still very close and very fusing, and everything that goes with it […]. And besides, I had a lot of comments about that, like, “It must be too good to be this close to your mom like that, you're too lucky.” I thought it was cool!”*

*Q43 Mother 2: “We talk a lot with Teenager 2 [.] the fact that she is experiencing this brought us together a lot and that we communicate a lot.”*

Q44 Teenager 1: “I, initially, I was good. Then it was complicated with Teenager 6 and Teenager 4 because there was some trouble and everything. So that made me a little bit sick of the stuff.”

*Q45 Mother 1: “Ah yes, her psychologist, yes.”*

*Teenager 1: “My model of a woman. Sorry mom!”*

*Teenager 1: “My second female model.”*

*Q46 Father 3: “Last session, I think everyone would have liked, not to have others sessions, but at least having the possibility to keep being guided by you occasionally. Because everyone seemed to feel we were now let down.”*

*Q47 Mother 6: “So far, we haven't done anything. Maybe someone needs to provide the impulse.”*

### The Living Experience of MFT

#### Description of the MFT Device by the Participants

##### Experiencing Generational Diversity Safely

The families were very sensitive to the fact that MFT mixed two generations. They valued that different points of views could be expressed with confrontations, disagreements, and debates (Q1). This diversity allowed a horizontalization of the relationships and gave a voice to the adolescents (Q1). Adolescents thus became experts of their own disorder, with a modification of the usual family dynamic. Parents saw their children from another perspective, in “real condition” with other adolescents (Q2). MFT brought also a diversity of roles with the sub-groups, adolescent sub-group, mothers sub-group and fathers sub-group, allowing a better cohesion, providing different expressions and pooling different resources. Diversity was also illustrated by families who were at different stages of school refusal and reflection (Q3).

Q1 Teenager 1: “It creates debates too. To see that sometimes we have as much voice as adults. It gave us confidence, whereas it's sometimes what we are blamed at high school for or that kind of thing. There, it feels good to be at the same level.”

Q2 Mother 5: “But in the end we have never experienced a situation like this, to be in a group, where you talk about a theme. I also discovered my daughter.”

Q3 Mother 6: “We were a bit of neophytes on the subject, whereas there are people who had thought about it a lot more.”

##### Getting Involved in Activities

Adolescents and parents emphasized five activities: problem drawing, sculpture, photolanguage, role-playing, and imaging the future with masks accessories. These medias expressed what speech couldn't. Serious things and emotions were shared by an imaginary and symbolic language (Q4). These activities made participants think and question themselves, but in a twisted and playful way (Q5). These exercises must be done by both parents and teenagers in order to compare different perspectives. Exercises put parents and teenagers on an equal footing (Q6). Parents perceived the experience of their teenager both through the activities and their explanation by adolescents. This was very striking for adults and adolescents, whose speech became strengthened, legitimized and shown. Their suffering, their experience was no longer heard but watched, furthermore by their parents (Q7). Through this look, it became real. In contrast, some activities were utterly not named, as the blazon one which worked on family resources, and the backpack which worked separation issues.

*Q4 Teenager 5: “By activities, we really can express things otherwise than with words. Precisely, to bring out things that come from our imagination and all that, it's something that brings out the real emotions much more.”*

*Q5 Teenager 4: “I would say we don't have too much to think about. Finally it made you think but not too much and it was still a pleasant moment.”*

*Q6 Father 6: “The moment when, symbolically, the problem was drawn by the parents on one side, by the children on the other [.] both sides of the same coin that really highlighted the different vision of the same problem.”*

*Q7 Teenager 1: “It felt good to show to parents, to put our fears into words.”*

##### Belonging to a Group

Group was noticed by all the participants as a major and supportive element: MFT group, parents group, adolescent groups, and subgroups (Q8). Relationships happened once more. By its kindly holding, its motherly matrix, group enabled speaking, emotion releasing and emotion flowing (Q9). Adolescents expressed themselves with a single voice, almost in a single body; their voice became legitimized and powerful, heard by their parents (Q10).

Group was an entity whose members experienced the same things, belonging to a single and merging collectivity (Q11). For some adolescents, it was the first time they met others teenagers suffering from school refusal. The group had to be preserved whatever the difficulties were, in a group illusion. Violence or family conflicts had not their place. Participants were excluded if they didn't share this single thought (Q12).

*Q8 Father 2: “I think this is what makes this thing so strong, it's the fact that there is a group.”*

*Q9 Mother 6: “Maybe to be in a group too, maybe there are things we say differently. I think we don't feel the same when we are several as when we are alone. I think it's a good way to get some things out.”*

Q10 *Mother 2: “What really strikes me is their total agreement, they had the same living, the same feelings and that really strake me….because they didn't know each other, they've never seen each other and when they presented their activity well, they were always in unanimous agreement, and that really struck me.”*

*Q11 Teenager 1: “Yeah, we made a single unit in an hour, but before we didn't know each other and it was the perfect square.”*

*Q12 Teenager 1: “It was just that the affinities were made at the start and then it just got worse. Suddenly, we just weren't compatible […], she didn't come back to me.”*

##### Therapists as a Security Figure

Therapists were deeply invested. They settled the frame in a reassuring way (Q13). By their holding and their welcoming, emotions could be released. Unlike others therapies where therapists are more leading, they stepped aside for participants to become co-therapists (Q14). One mother expressed that if therapists had participated too much, she would have felt paralyzed (Q15). On the contrary, half of the parents found that therapists were not enough interventionists. These latter parents seemed to be looking for reassurance, security, confidence, and renarcissization of their parental or family functioning. It echoed parental regression toward the therapists. For them, therapists were the ones who got solutions, possessed the knowledge and knew the appropriate behavior parents should have (Q17).

*Q13 Mother 5: “I found that you have been able to create an atmosphere, for people to feel confident and that enable it […]. As a secure framework.”*

Q14 Teenager 3: “In the end, it guides us toward solutions, which leads us to do some thinking alone. It's still really interesting for us to lead the session a bit ourselves, still being guided not to go anywhere.”

*Q15 Mother 3: “I, if I may add, if you had intervened, personally, I would have stopped talking because we would have felt judged, analyzed.”*

*Q16 Father 3: “By the way, if I have a criticism to make, I find that you did not intervene much.”*

*Q17 Mother 6: “When you said: ‘we're filming,' I say to myself: wow, they must have analyzed things, they saw: there, he ticked, there, he did something. I, I don't know how to analyze.”*

#### A Deeply Emotional Trip

##### Emotional Catharsis

Participants described an emotional catharsis, feelings restrained which could eventually/finally be released in the group, because the group welcomed and greeted them (Q18). These emotions flowed in a dual circulation, the participants being both depositories and receptacles (Q19). Therapeutic work was enabled (Q20). Participants described an emotional range and thus experienced emotions once more (Q21). Even though speaking of and feeling these emotions was painful, participants explained that it was necessary. They were relieved and even proud of themselves (Q22). Intimacy was exposed Q23).

*Q18 Mother 3: “[…] and you are given the opportunity to open the floodgates, that was my thing. There you are in a group where everyone has the right to express themselves. So automatically, barriers you put up to hold out fall.”*

*Q19 Mother 3: “So there were a lot of emotions, often it was a parent and then at another session another parent. It was spinning.”*

*Q20 Father 6: “Once again, group time is therapeutic, emotional, it works.”*

*Q21 Teenager 5: “We went through all the emotions, I would say between us.”*

*Q22 Father 3: “I was struggling and still struggling. It is true that it took me to the guts. But I felt it was necessary.”*

*Q23 “Mother 2: “we bare with people we don't know.”*

##### Group Connection

Participants experienced deep connection between each other. They felt strongly connected in their experience of the difficulties (Q24). Some personal history echoed strongly between parents and teenagers (Q25). In addition, some participants used the word mirror: mirror as a reflection which enabled a questioning, and mirror as a multiplier medium that strengthened the therapy work (Q26).

*Q24 Mother 2: “Compared to what others might experience, feeling their experience [.]. I had resonance with some people.”*

*Q25 Mother 1: “My problem was uprooting […] it was Ado 5 experience, which also upsets me a little [.] to be like that, cut off from its roots, of its origins.”*

*Q26 Father 3: “Because there is a mirror effect, that is to say that we see other parents playing teenagers, we see teenagers playing parents and there, we understand things better.”*

*Q26 Mother 5: “Me, I would say that it has a mirror effect, it multiplies. Instead of seeing only you in the mirror […]. So that's just as much power.”*

##### Awareness

Parents described a true awareness of their teenager experience (Q27). It was a revelation of their adolescent autonomy, maturity and lucidity (Q28). Some parents even spoke of bewilderment. This awareness was seen in certain terms used, such as “struck,” “marked,” “questioned,” “percussive.”

*Q27 Mother 4: “This stress, this look is so deep, I hadn't understood enough before, this fear to make a mistake, the look of others, it is so strong. I understood that a lot more with the multi-family group. I hadn't realized that.”*

*Q28 Mother 4: “It was really interesting and with a clarity, a clarity… Yes, I think we were all stuck, in any case the parents, I find we were much more draft, whereas they were very clear, precise, they knew where they were going, they really have an awareness of what they are going through and they know how to put words.”*

### After MFT: Outcomes and Unmet Expectations

#### Empowered Families

MFT was an extremely invested therapy by the participants (Q1). MFT partly relieved parents and adolescents from their guilt (Q2). It offered them other ways of proceeding and solutions (Q3). They better understood school refusal. Going back to school was no longer a goal for some parents (Q4). Intra-family communication was improved (Q5). Participants understood that part of the solution was inside the family system (Q6).

*Q1 Father 3: “I didn't think MFT would offer me so much.”*

*Q2 Mother 5: “Yes, I also told myself that I had a part of guilt I could leave.”*

*Q3 Mother 3: “We hear other ways of acting, saying: well, maybe I will try, it will maybe improve things, help him a little more. We found other solutions finally*.

*Q4 Father 3: “Back to school? I had it before, now I understand that it's not necessarily .”*

*Mother 3: “We accepted the situation. We even told Teenager 3: “Going to school might be not worthy if that makes you feel stressed””*

*Q5 Father 3: “We question ourselves. I listen more to my daughter, I try to listen to others a little more, I see that she has less difficulty speaking, even with us.”*

*Teenager 3: “It also allowed more communication.”*

*Q6 Mother 4: “I don't know, it's not individual, it's not a case, a single case. I think it's interesting in itself to see other families. For me, it's a structure. If I move, things will also change with Teenager 4 or the opposite. In any case, we are not isolated individual, everything is connected.”*

#### More Liberated Adolescents

MFT brought adolescents a liberation and a relief (Q7). Adolescents experienced a certain authenticity in relationships in MFT thanks to the absence of peer judgment and therefore experienced a peaceful relationship with their peers (Q8). They had more confidence in themselves (Q9).

*Q7 Teenager 5: “It allowed me to open doors, to be less stuck on school refusal or even anxiety, things like that.”*

*Q8 Teenager 5: “I felt comfortable, not misunderstood. I had less this feeling of wanting to be superior to others […]. I felt in my place […].”*

*Q9 Teenager 1: “You are going to go to a group where there will be lots of people like you. Suddenly, it gave us confidence, we are able to do it.”*

#### Breaking the Taboo of School Refusal: Acceptance, Talk About

School refusal was finally accepted and told to others (Q10). Participants have been able to talk freely about it. MFT provided them answers (Q11). Participants admitted school refusal (Q12)

*Q10 Mother 3: “Me, I hadn't told my parents. There, I said to myself: now you're going to say it and then we'll see.”*

*Q11 Teenager 5: “I think it helped me on a lot of questions I was asking myself”*

*Q12 Mother 2: “She's going to be able to tell it even to people she doesn't know.”*

#### Relationships With Others: Socialization, Communication, Differences

Participants felt less lonely (Q13). Absence of judgment and sympathy were two major elements (Q14). MFT allowed to have new contacts and to face others perspectives in safety. The community and its plurality of perspectives helped each other (Q15). Thus, each participants became co-therapists. Moreover, seeing others reaction made some participants question their behavior and change it afterwards (Q16).

*Q13 Mother 5: “It already made it possible to see that we were not alone. It's comforting to know that we are not alone facing things.”*

*Q14 Mother 3: “Not to be judged. This is also what is important, no one judges the other.”*

Q15 Father 1: “We are all together with our own problem, helping each other to find a solution together.”

*Q16 Father 3: “It's the mirror phenomenon, I said to myself: hey, the guy doesn't have a good attitude. And then I understood that I was doing the same.”*

#### Unmet Expectations

Parents didn't see results about school refusal (Q17). They would have liked more practical solutions to be provided. Some families would have liked feedback from therapists, and even made it one of the goals of MFT; they missed feedback and interventions (Q17).

*Q17 Mother 5: “I haven't seen too many effects on ‘Avoiding chronic school leaving' and ‘Improving anxiety manifestations'.”*

*Q18 Mother 6: “I found that it lacked feedback.”*

## Discussion

The aim of this study was to explore the experiences of adolescents and their parents of the MFT in school refusal. We aimed to explore the adolescents and their parents perspectives, the relevant improvements and the unfulfilled expectations. We explored reported changes on individuals and intra-family interactions. We also studied if what was described in MFT theory was found through families reported experiences, and if it was relevant for school refusal treatment.

### Families Experienced MFT as a Therapy Between Family Therapy and Group Therapy

Our results suggest that MFT provides families with reassuring environment, group connections, empowerment and various perspectives. This echoes several aspects of systemic family therapy.

One of the key objectives for family therapists is to “join” the system in order to be able to work with its members. To do so, they must understand and respect the rules and organization of the family system (48). In our results, therapists joined the multifamily system by understanding the implicit rules of the group and of its family sub-systems- including the fear of direct confrontation, either to psychiatry, other people, or to their own emotions. Moreover, each participant affiliated each other, thus strengthening the joining procedure.

Moreover, our results suggest that the resonance phenomena occurred and was particularly strong between participants, who thus became co-therapists. Resonance states that, in a system, one member's experience- toward an emotion or an event shared or expressed in it- has a function for the system (49). The resonance phenomena is part of the family therapy.

Further, MFT seems to use the family competence as a therapeutic tool. Family competence is a concept developed by G. Ausloos who states that families have already the solutions to their problems, but they do not know it yet. Therapists only guide families to find it (50). In our study, participants not only valued their own family unit's competence but also the competence of other families. The group appeared as a larger family, where each family unit was able to provide solutions. In addition, according to G. Ausloos, families became active agents of their own change. They are included in the therapeutic process and relieved from their guilt. In our results, the participants described those two specific aspects.

Finally, in some families, communication has reached a dead-end: the same interactions are repeated over and over, in an endless game. Each apparent solution fails to provide a real change, “doing more of the same thing” (51, 52). Family therapy, through therapists, brings other new perspectives (53). Our results suggest that metacommunication was occurring in our study. Each family brought another perspective and became thus co-therapists. This change of perspectives and roles is described by E. Asen as a “greenhouse effect”: participants are “in perpetual motion” within their family and the MFT group, having to adopt a multitude of roles and perspectives (34).

Families included in the study also underlined several aspects of group therapy, such as activities, group experience as a belonging place and a safety framework, and eventually mirror reactions.

Media such as activities, drawings, role playing and sculptures enables adolescents to make up with their thoughts passing by imagination (54). Indeed, while the blank sheet of paper astounds, media allows liberation. In ours study, imagination- so crucial to the transformation process occurring at adolescence- was paralyzed by school refusal and was particularly unleashed by activities (55, 56). Furthermore, through activities, adolescents were at the same level as their parents, in this horizontalization process allowed by MFT. By using their language and their expression code, MFT allowed their integration and involvement into the therapeutic process. Their words became heard. They became therapists, experts of their disease and valued. They, who suffered from school refusal and anxiety, deadlocked in daily life and in their families, became active agent of their change. They reappropriated their thinking from their parents who all expressed their surprise facing the lucidity of their teenagers's words. MFT thus allowed progressive empowerment of thought and differentiation from parents.

Further, the group gave to adolescents a new and transitory support of identity (57). The sense of belonging to this “perfect square” allowed them to face anxieties of fragmentation and of identity loss. The group was the basis of a neo-identity, a “prosthesis” which helped supporting their failing feeling of their own personal identity (58). Shared perception of schooling or of school refusal provided support to face differences. Adolescents could give up some defensive attitudes (59). As Teenager 5 pointed out, MFT became a place where she could be more genuine without trying to be superior. Adolescents could re-experiment the community experience and interactions with peers. They surrendered their social anxiety, their fear of judgment and their experience of exclusion.

Moreover, the group device, with its frame and sympathy, held emotions and anxieties (60, 61). *Holding* was also set by therapists and their special positioning in group therapy. Indeed, they did not have the classic leader role and acted as benevolent observers who provided care function- as “co-pilots”- making families active agent of their own change (34, 62).

Last, the mirror reaction was widely noticed by participants. Foulkes describes it as the patient's awareness that other individuals have morbid anxieties, which allays anguish and guilt (62). Mirror reaction also includes the personification, the incarnation of a character in which participants can recognize themselves or discover- through their reactions to him- an unknown part of themselves.

### MFT: A Relevant Therapy for School Refusal

Between family therapy and group therapy, MFT appears in our results to be truly relevant for school refusal management.

Emotions, experiences and feelings could be expressed in families where they were precisely locked in. Emotions and speech were eventually released and flowed. This emotional update created a real “sensitive period” mobilizing new representations of oneself, of others, and of the interactions in the family (63). MFT restored moves and circulation in families paralyzed with an injunction of impossible motion. This flow of emotions even went beyond the intra-family nucleus to outreach the extended family, as exemplified by Family 3 whose maternal grandparents “evolved.”

Emotion was released in safety, allowed by MFT device. By the frame, the holding, the therapists positioning and the activities, intimacy could be shared without threat. The benevolent needs of adolescents were thus fulfilled.

In addition, for these families characterized by separation anxiety, working precisely in family was probably a major element, enabling autonomy in safety. Adolescents could be with peers under their parents “look” who saw their offspring evolving in a micro-society of peers. As noticed by Mother 3, it was the first time for many parents that they experienced their adolescents evolving with other teenagers.

Besides, in MFT, attachment issues, attachment to the therapists and to the other participants occurs regularly. J. Byng-Hall defines the concept of “family security basis” which allows in family clear communication, open feelings expression and the ability to recruit outside help from extended family, as resource persons or therapists (64). In MFT, the therapists and other participants became substitutes and temporary family security basis in this group, which became a meta-family (65).

It is interesting to note that the positioning of the therapists confused and made half of the parents felt insecure. The end of MFT was difficult for all parents, as if the separation was impossible. But, as a paradox, seeing each other after MFT could not happen, as if the transition to reality was impossible. We can hypothesize that if the group is therapeutic as an identity, seeing itself outside would be too intrusive with a threatening reality.

Further, for these families who experienced exclusion and stigmatization, MFT allowed enrollment in a system of peers: group system, school refusal system, MFT system, adult system, teenagers system. Rejected by “a common enemy,” searching for a validation and a legitimacy of their abnormality, participants found in MFT a place to belong. It enabled them to go back to the institution, whether for an individual therapy, a family therapy or day hospital care.

Finally, MFT works on belonging and identity issues in a system where filiation and affiliation axis co-exist, which is unusual (66). This appears all the more relevant for this problematic which itself is not recognized as a psychiatric disorder and which does not clearly belong to a diagnostic entity “without affiliation, without socialization, and almost without perception of its limits [of existence] (66).”

### School Refusal Gets Health Professional to Work With Community

Very few participants, especially parents, clearly used the term of school refusal. They spoke of problems, anxious refusal, and social anxiety. Similarly, they neither talked about depression nor unease. While for other psychiatric disorders, adolescents consult a doctor to recover, here solution should come from school. While adolescents came to MFT for their parents to understand their disorder, parents went to MFT in order to help their children, wishing a return to school after the therapy. This difference of expectations recalls Sibeoni study (25), where adolescents wanted peer relation and going back to education more than going back to school.

The specific place of this disorder can be seen in family ambivalence toward school refusal- which marked an identity, an extra-ordinary identity. It is also seen in MFT expectations, which should bring “educational guide.” We could wonder whether the difficulty was the school refusal or the inability of the academic system to adapt to these “extra-ordinary” pupils. Parents thus seemed to come to MFT and to the hospital, another state institution, in order to find the help and support that school was unable to provide them. A cleavage appears between the hospital, the good one, and the academic system, the bad one, in a disorder at the crossroads of these two entities. MFT became “a last resort” and carried the unfulfilled expectations.

Further, as we introduced, school refusal does not yet appear in international classification, neither in the DSM 5 (Diagnostic and Statistical Manual of Mental Disorders) nor in the ICD-10 (International Classification of Diseases). If the DSM-5 includes school refusal in “anxiety disorders of childhood and adolescence linked to separation anxiety” and distinguishes two forms- the first classified among the symptoms of separation anxiety and the second classified in within social phobia (DSM-5)-, the ICD-10 classifies this disorder as “phobic anxiety disorders” (ICD-10). It seems to suggest that psychiatry community considers that school refusal is a social functioning disorder, explained by social and academic reasons. Besides, for parents, school responsibility is often essential if not exclusive and the care offered by the psychiatrist enters into competition with the return to school and to normality (54).

These results raise several questions. First, what would mean a return to school: a recovery? A return to normality? Would it be a criteria for the recovery of a psychiatric disorder? Can it really be a therapeutic goal? It highlights the question of care and its limits- in families where it is probably easier to talk solely of school issues.

Furthermore, this study questions the place of education in families and, more broadly, in society itself. The solution must consider this societal dimension, and first and foremost this third protagonist, school. It seems necessary either to invite the school to MFT or to go to meet the school. We thus better understand parents' expectations from MFT, which appear at first sight unsuitable to MFT (bringing concrete solutions, making the link with the academic system).

From the psychiatric community to the “network therapy,” working with- even in- the community is an important issue in psychiatry. In the 1970s, Cooper (67) founded the Philadelphia Association for developing original places, as Kingsley Hall, for patients suffering from schizophrenia. Patients were taking turns for caring for each other, the group regulated itself and controlled the delirium of its members. Likewise, Speck (68) developed a new professional role, a mediator one between the schizophrenic family and the therapist, between the therapist and social organizations or society, and between family and society. “Network therapy” uses the patient's network for care, a network in the broad sense (which can go up to 40 people): nuclear family, extended family, friends, and neighbors (68). The “Family School” developed by Dawson and McHugh's team at Malborough Hospital falls into this tradition in order to work with, even in the community and not in the institution (42, 43). In France, Prof. Baleyte, head of department of the 5th sector of child and adolescent psychiatry at the intercommunal hospital of Créteil, has developed since 2018 a mixed and mobile school intervention unit, the UMMIS, which relies on the tools of multifamily therapy (69). UMMIS is a multidisciplinary mobile team that works in several schools in the city of Créteil to prevent from dropping out of school. It relies on the commitment of three stakeholders: the school, the pupil and his family, and UMMIS.

We could thus reflect on the opportunity of such a device in the management of school refusal which would include families, teachers and therapists. One of the longer-term objectives would be to develop a joint network with education in order to be able to quickly take care of patients suffering from school refusal who often arrive too late to the care.

Finally, this study only included a few sessions, and the beneficial effects could be impacted. More sessions have been added to the next ones (8 sessions). The results of this exploratory study were considered to improve the content and form of the following groups. Other groups will start in other units and we hope to be able to consolidate the results.

### Strengths and Limitations

Our study had several strengths. First, to our knowledge, this is the 1st study, and the 1st qualitative one, exploring MFT experience of families facing school refusal in adolescents. This was the first group of MFT and school refusal organized in France, making this study a pioneering one in that field. Moreover, the rigorous IPA-based analysis was most appropriate to its topic.

Nonetheless, some limitations must be taken into consideration.

First, it took place in France, and caution is required in transposing our results to other places because psychiatric care depends strongly on the organization of the medical system as well as on the country's economy. Second, the population of adolescents was recruited in a specialized department of adolescent psychiatry.

One might argue that the sample size is too small to allow transferability of the results and that data saturation was not reached. However, this small homogenous sample is in line with IPA guidelines, and the concept of data saturation is not relevant within IPA methodology (46).

One methodological limitation is the jointly participation of adolescents and their parents during the research interviews which might have inhibited adolescent expression.

Finally, as only one brother came to only one session, we didn't include the siblings in the research but further research should include their perspectives.

## Conclusion

This research highlights several elements.

On one hand, families' experience of MFT seems to confirm its anchoring in both group therapy and family therapy. Indeed, it takes root in family therapy, as it leverages systemic concepts such as resonance, affiliation, family competence and metacommunication. It also borrows from group therapy the use of a medium, the group device- which composure help create a safe environment-, and the role of therapists- who leave the participants active agent of their change. The MFT group thus becomes a large system, a meta-family whose members, individuals or families, become co-therapists, disorder experts and agents of change, by mobilizing their family or even meta-family resources.

On the other hand, MFT appears to be quite relevant for school refusal management, a paralyzing pathology, where emotions and thoughts cannot be expressed. It allows working on separation issues in families where it seems difficult to empower oneself. In addition, it is a pathology at the crossroads of care and education, and which gives rise to great stigma. MFT group thus appears particularly relevant since it offers participants a new affiliation and a relief from their guilt.

It is interesting to note a certain resonance between school refusal and MFT since they both are two fairly recent entities whose identity is not clearly defined.

Finally, this study questions the role of school in therapy. MFT has its origins in community psychiatry and it seems important to have a reflection on how we can work with this third protagonist in order to fulfill families' expectations for a disorder that appears to be deeply rooted in society.

## Data Availability Statement

The raw data supporting the conclusions of this article will be made available by the authors, without undue reservation.

## Ethics Statement

The studies involving human participants were reviewed and approved by Comité d'évaluation éthique des projets de projets de recherche en santé non soumis à CPP N° IRB: 20151300001072. Written informed consent to participate in this study was provided by the participants' legal guardian/next of kin. Written informed consent was obtained from the minor(s)' legal guardian/next of kin for the publication of any potentially identifiable images or data included in this article.

## Author Contributions

The study was designed by AH. The interviews were independently coded by two researchers (AR and AH). Codes were discussed during group meetings (AR, AH, LB, JS, and MM) to enable triangulation, which both enriches the analysis and serves as a quality control process. All authors contributed to the article and approved the submitted version.

## Conflict of Interest

The authors declare that the research was conducted in the absence of any commercial or financial relationships that could be construed as a potential conflict of interest.
